# The Influence of Lath, Block and Prior Austenite Grain (PAG) Size on the Tensile, Creep and Fatigue Properties of Novel Maraging Steel

**DOI:** 10.3390/ma10070730

**Published:** 2017-06-30

**Authors:** Thomas Simm, Lin Sun, Steven McAdam, Paul Hill, Martin Rawson, Karen Perkins

**Affiliations:** 1 College of Engineering, Swansea University, Swansea SA1 8EN, UK; steven.mcadam@element.com (S.M.); K.M.Perkins@Swansea.ac.uk (K.P.); 2 Department of Materials Science and Metallurgy, University of Cambridge, Cambridge CB3 0FS, UK; permax@gmail.com; 3 Rolls-Royce PLC, Derby DE24 8ED, UK; Paul.Hill2@Rolls-Royce.com (P.H.); Martin.Rawson@Rolls-Royce.com (M.R.)

**Keywords:** creep, strength, ductility, fatigue, martensite, electron back-scatter diffraction (EBSD), maraging steel

## Abstract

The influence of martensitic microstructure and prior austenite grain (PAG) size on the mechanical properties of novel maraging steel was studied. This was achieved by looking at two different martensitic structures with PAG sizes of approximately 40 µm and 80 µm, produced by hot rolling to different reductions. Two ageing heat-treatments were considered: both heat-treatments consisted of austenisation at 960 °C, then aging at 560 °C for 5 h, but while one was rapidly cooled the other was slow cooled and then extended aged at 480 °C for 64 h. It is shown that for the shorter ageing treatment the smaller PAG size resulted in significant improvements in strength (increase of more than 150 MPa), ductility (four times increase), creep life (almost four times increase in creep life) and fatigue life (almost doubled). Whereas, the extended aged sample showed similar changes in the fatigue life, elongation and hardness it displayed yet showed no difference in tensile strength and creep. These results display the complexity of microstructural contributions to mechanical properties in maraging steels.

## 1. Introduction

Maraging steels are a class of ultrahigh strength steels that consist of a martensitic microstructure hardened by intermetallic precipitates. They have found use in a number of industries most notably in aerospace due to their combination of high strength and damage tolerance. In a separate paper on maraging steels [1] the importance of the choice of ageing heat-treatments in producing different precipitate populations was described. It was shown that significant mechanical property improvements could be made by the choice of austenisation temperature: a creep life increase of over three times, strength increase of 150 MPa and ductility increases of over three times. However, it was found that there was a trade-off between ductility and the other properties. Grain size refinement by thermomechanical processing has a potential advantage over ageing treatments in that it has been found that it can improve a range of mechanical properties at the same time, including ductility, strength, creep and fatigue. The work of Petch [2] based on the experiments of Hall [3] established the importance of the grain size on the strength of an alloy. The relationship between grain size and a range of mechanical properties have been modelled and measured, including strength [2,4], ductile to brittle transition [5,6], creep [7,8] and fatigue [9,10]. The majority of this work has been based on alloys with an equiaxed grain size, but with maraging steels there is an added complication of multiple definitions of what constitutes size since a martensitic microstructure consists of a number of prior austenite grains (PAG) each of which consist of packets, blocks and laths. A packet is a region that consists of a number of parallel laths with the same habit planes. These laths can either be slightly misorientated or have very different orientations from each other, whereas a block is a series of laths with similar orientation. The use of orientation mapping by EBSD has allowed a greater understanding of the martensitic microstructure and allowed the determination of block sizes that is not possible to determine by standard microscopy [11,12]. This understanding of block sizes has led some researchers [13] to conclude that the block size, and not the packet or PAG size (as previously thought), is the controlling size element in the yield strength of a martensitic alloy. Ageing heat-treatments will change the strength of the alloy and can embrittle it, however they are not expected to influence the relationship between strengthening and grain size [14]. In this paper, we consider how the microstructure influences the mechanical properties of a maraging steel by considering two different PAG sizes and two different heat-treatments. One is aged for 5 h and the other has an extended age at a lower temperature.

## 2. Material and Methods

**Figure 1:**
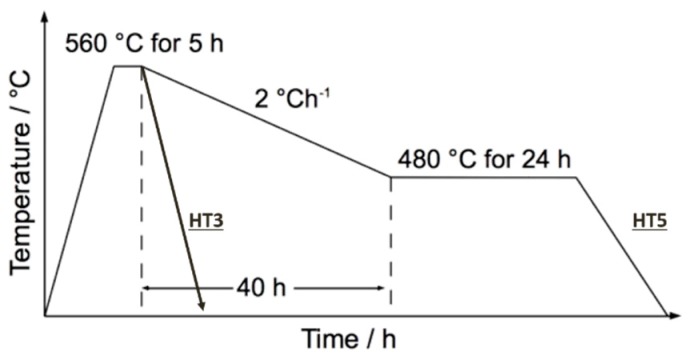
Schematic of the two ageing heat-treatments.

The composition of the alloy is shown in Table 1. The alloy was vacuum induction melted by Tata RD&T Swindon technology centre as 60 kg ingots (~150 mm × 150 mm × 410 mm). They were hot forged to produce two different PAG sizes which will be named based on the size of the final bar: 50 mm and 19 mm. The 50 mm bars were hot forged to 50 mm × 50 mm square bar and homogenised in a vacuum at 1200 °C for 48 h followed by gas quenching using high purity Argon. The 19 mm bars were produced by hot forging these 50 mm bars. The two bars were then aged to two different heat-treatments, HT3 and HT5. HT3 consists of an austenisation at 960 °C for 1 h and ageing at 560 °C for 5 h followed by an air cool. HT5 is the same but with an extended slow cool to 480 °C over 40 h, then aged at 480 °C for 24 h and then air-cooled (Figure 1). Following heat treatment, tensile, creep and fatigue specimens were machined.

Mechanical testing was carried out at SMaRT (Swansea University, Singleton Campus, Swansea, UK). Tensile tests were conducted with a dual strain-rate of 0.00025 s^−1^ up to 2% proof stress and 0.068 s^−1^ thereafter, using the standard BS EN ISO 6892-1:2016. Creep testing was carried out by applying a constant load to the sample, at a temperature of 500 °C following the standard BS EN 10291:2000. Both creep and tensile test pieces were the same size with a gauge length of 20 mm and a diameter of 4 mm. Load control fatigue tests were conducted using the standard ASTM E466-07 with a trapezoidal waveform with a frequency of 0.25 Hz and R = 0. The test pieces used had a gauge length of 12 mm and a diameter of 4.5 mm.

EBSD maps were obtained using a Phillips XL30 SEM (Swansea University, Swansea, UK, operating at 20 kV with Oxford Instruments HKL camera and Channel 5 software). Maps had an average indexing rate of more than 80%, and were subsequently cleaned to remove non-indexed points. A step size of 0.5 μm and 1 μm was used for PAG and block determination and a step size of 0.1 μm for laths. For the determination of PAG and block sizes several maps were obtained with an area of approximately 0.5 mm^2^ for the 19 mm bar size and 1.5 mm^2^ for the 50 mm bar size. For the determination of lath sizes an average of 11 maps of 30 × 30 μm was used for each sample.

## 3. Results

### 3.1. The Martensitic Microstructure

#### 3.1.1. Methodology

In maraging and low carbon steels the orientations of blocks within a PAG have distinct orientations, called variants, due to orientation relationships (ORs) between the austenite and the transformed martensite. The three main ORs in steels are Nishiyama-Wassermann (N-W) [15], Kurdjumov-Sach (K-S) [16] and Greninger-Troiano (G-T) [17]; with different researchers suggesting that different ORs are more suited for different low carbon steels [11,18] or that all are an approximation because the analytical solution is irrational and different to these [19,20]. Laths and packets can be determined from etchants that reveal the lath boundaries. But blocks cannot be determined in this manner and instead they can be determined by EBSD scans. PAGs can in most cases be determined by etching, such as when precipitates or elements segregate to them, but this was found to be problematic for this alloy and so the use of EBSD offers an alternative method. PAGs cannot be revealed by standard misorientation boundary maps, i.e., by plotting boundaries above a certain angle, as misorientations between blocks are expected at a range of distinct angles. Instead, the misorientation angle and axis needs to be considered to reconstruct the PAGs based on a particular OR using the EBSD data [21,22,23]. There are different approaches, but they are all based on constructing probable PAGs based on the orientation of blocks, or individual orientations, and their position in relation to a chosen OR. In this work we use the program ARPGE [21] to reconstruct the PAGs. The G-T OR is used because it best matches the misorientation profiles.

The choice of what constitutes a block is non-trivial but can have important implications when compared with the mechanical test results. From Table 2 it can be seen that if a block is defined as misorientations greater than 5°, the ratio of block-sizes of the two bars is less than if the higher 15° misorientation is chosen. From the G-T OR, block boundaries can have misorientations at 3.5°, 7° and 11° in addition to ones at higher angles. However, we will define the block size here from misorientations above 15°, which would be larger than some of the possible variants. The value is chosen because boundaries with small misorientation are easier for dislocations to cross [24]. Boundaries of above 2° are attributed to lath boundaries, to reflect a slight misorientation across boundaries of laths of the same variant and to limit errors involved in determining the orientations. Both the block and PAG sizes are determined from the linear intercept method.

#### 3.1.2. Microstructure

**Figure 2:**
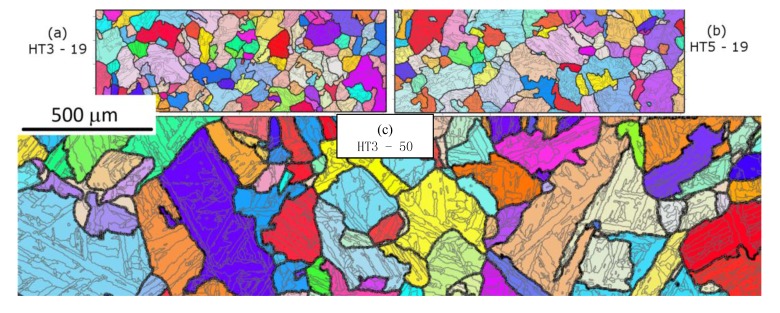
EBSD maps of the different starting microstructures. (**a**) HT3 19 mm bar; (**b**) HT5 19 mm bar; (**c**) HT3 50 mm bar. The maps display boundaries of different types the thick black lines are used to represent probable PAG boundaries (calculated by ARPGE), thinner grey lines represent block boundaries, and are boundaries with misorientations greater than 5°. The colours represent the IPFz orientation of the austenite as calculated by ARPGE. All figures have the same scale, shown on the left.

**Figure 3:**
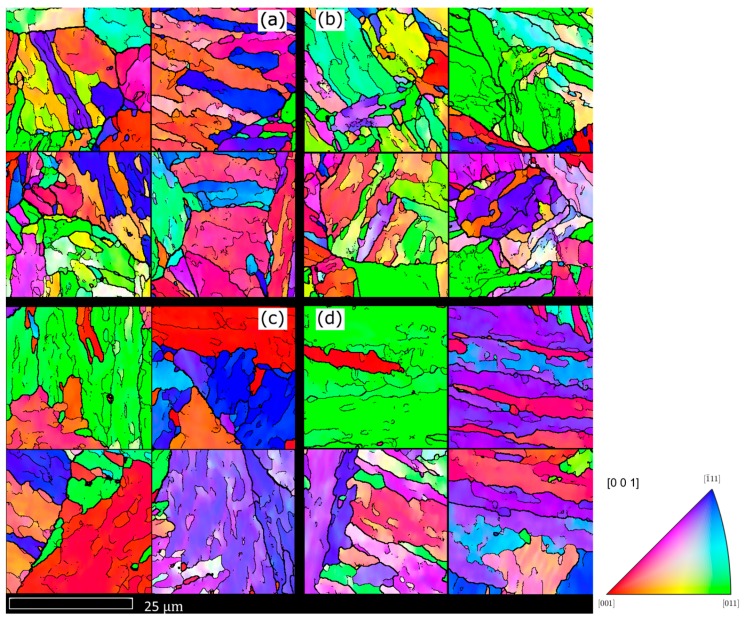
EBSD maps with IPFz colouring of the different microstructures. (**a**) HT3 19 mm bar; (**b**) HT5 19 mm bar; (**c**) HT3 50 mm bar; (**d**) HT5 50 mm bar. The single pixel black lines represent boundaries with misorientation between 2° and 15°, 2-pixel black line between 15° and 21° or more than 48°, and 3-pixel black linesbetween 21° and 48° to represent probable PAG boundaries. The maps are used to determine the lath sizes in Table 2.

After thermomechanical processing and heat-treatment the alloy displays typical martensitic microstructures found in maraging steels (or steel with low C) [11,12] as shown in Figure 2 and Figure 3. The microstructure is fully martensitic and consists of PAGs, packets, blocks and laths. The blocks are mostly elongated and in some cases extend across the width of a PAG.

The microstructure of the 50 mm bars is coarser than the 19 mm bars, with larger blocks, PAGs and laths as shown in Table 2. The PAG size is approximately twice larger, the block size 40% larger and the lath size 26% larger for the 50 mm bar size than the 19 mm bar. Whereas, the two heat-treatments have similar sizes for the two bar sizes. Based on work from other researchers on steels [6,12,25] a relationship between the size (*d*) of PAG and blocks can be expressed as:

(1) $$d_{block}=\eta d_{PAG}^{1/2}$$

**Figure 4:**
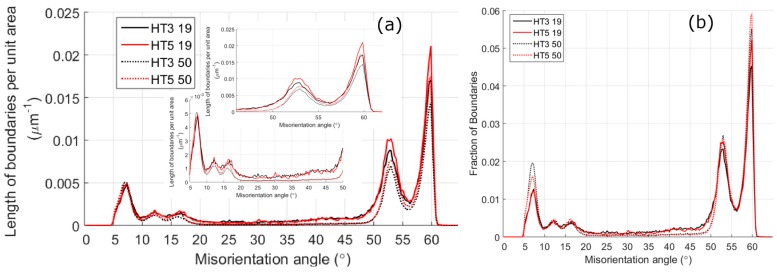
Misorientation angle profiles calculated by MTex for grains with misorientation angles above 5°, for the different samples studied. (**a**) Are the length of boundaries (in μm) per unit area (μm^2^) (normalised for different map sizes); and (**b**) the fraction of different boundary types.

For the alloy studied we find this same relationship exists but with a value of *η* of 1.2, whereas for a combination of HSLA, maraging and low carbons alloys accumulated by [26] 0.3 were found. This means our alloy has fewer blocks per PAG (or bigger blocks for a given PAG size), which may be partly due to there being significant numbers of block boundaries less than the 15° chosen here to define a block boundary. Based on the results in Table 2, a similar relationship appears to exist with the lath size, but with an exponent between (1/3) to (1/4) for the PAG size to reflect the smaller change seen in the lath structure between the 19 mm and 50 mm bars, as seen in Figure 2 and Figure 3. The misorientation angle profiles for the different samples are shown in Figure 4 and the number of boundaries between different angles is shown in Table 3. These profiles are consistent with the size values of the different samples shown in Table 2; they show the 19 mm samples have more boundaries per μm^2^ than the 50 mm samples at most angles, and hence smaller block and PAG sizes. The figure also display that the two heat-treatments have similar misorientation profiles and hence lath structures, albeit as shown in Figure 4b the fraction of different boundary type differs. For those boundaries between 20° and 50°, representing mainly PAG boundaries, there are approximately four times more boundaries for the 19 mm bars. At other misorientation angles the difference in the number of boundaries in the two bar sizes is reduced; there are approximately 40% more boundaries in the 19 mm samples at angles around 15° and 52° and the difference is negligible at misorientation angles less than 10° and above 58°. These differences may suggest that certain boundaries between adjoining variants become more favourable with increasing PAG size. For the lower angle boundaries the difference may instead be a consequence of some of the boundaries being lath boundaries, rather than different variants; this is because lath size have a smaller dependence on PAG size than block boundaries.

The martensitic microstructure is similar to cold worked metal being heavily distorted with a high dislocation density [27]. When deformed metals are subjected to elevated temperatures the microstructure can transform by grain growth, recrystallisation or recovery; the last of these consisting of an annihilation of dislocations, formation of low angle boundaries and subgrain growth. There is some difference between the response of cold worked and martensitic microstructure at elevated temperatures [28], and a martensitic microstructure found to be more stable. The similarity between the microstructure of HT3 and HT5 shows that the lath microstructure is fairly stable at 500 °C, which is consistent with the work of others [28,29].

As with other maraging steels, considerable strength is gained by ageing this alloy to produce a dispersion of precipitates. More details of the precipitates and their impact on the properties of this alloy can be found in a separate paper [1]. The alloy is strengthened during heat-treatment by small NiAl precipitates (~5 nm) and larger laves phase (rich in W and Mo) precipitates (~20 nm). During the extended age of HT5 additional Cr rich precipitates are produced along with the growth and formation of new NiAl and laves precipitates.

### 3.2. Mechanical Properties

#### 3.2.1. Strength Results

**Figure 5:**
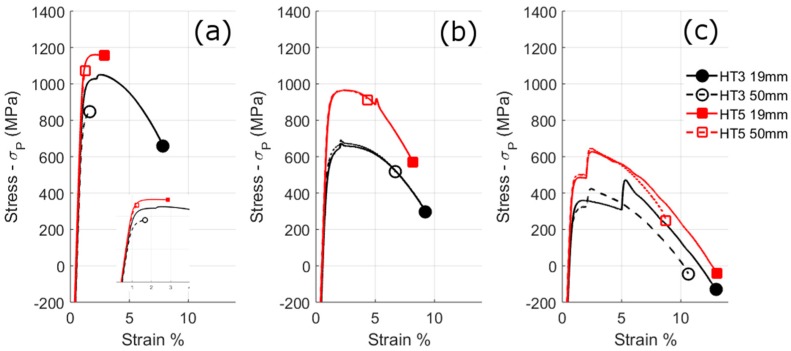
Tensile test stress-strain curves for HT3 and HT5 for the 19 mm and 50 mm bar sizes. *σ_P_* is the strengthening by precipitation of HT3. (**a**) at room-temperature; (**b**) at 200 °C; and (**c**) at 450 °C.

**Figure 6:**
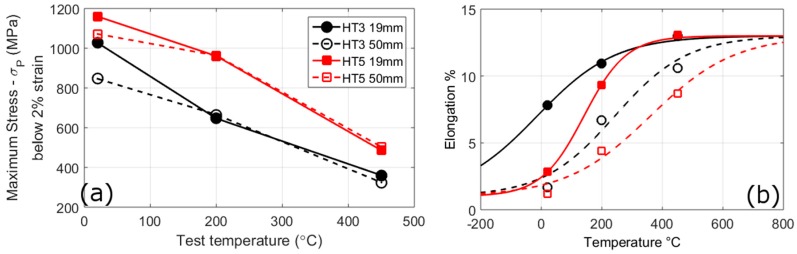
The change in yield stress (**a**); and elongation to failure (**b**) with temperature for the different conditions. *σ_P_* is the strengthening by precipitation of HT3.

The stress-strain curves of the different bar sizes and heat-treatment conditions are shown in Figure 5, the change in yield strength with temperature in Figure 6a, and the micro-hardness in Table 4. For the stress values the extra strengthening by precipitation of HT3 (*σ_P_*) is subtracted from the values.

The micro-hardness results show an increase in the hardness of HT5 compared to HT3. This difference though is much smaller than the difference in hardness between the two melt sizes. With an increase of ~200 HV between the 19 mm melt and the 50 mm melt (higher for HT5 than HT3), this would correspond to a strength increase of ~600 MPa using the expected conversion rates.

As with the hardness results HT5 has a higher tensile strength than HT3 at all temperatures. The stress-strain curves show a similar shape for the 19 mm and 50 mm melts, but there are differences in the yield and ultimate tensile strengths, and the elongations to failure. In contrast to the hardness results, the difference between the two melt sizes is much smaller. Whereas, HT3 shows a significant increase in the strength of the 19 mm bar compared to the 50 mm bar at room temperature of 167 MPa, for HT5 and HT3 at other temperatures the difference is much smaller and in many cases within the error of the measurements. The differences in strength of the bar sizes are much lower than found from the hardness results. The brittle fracture of some samples at room-temperature adds an uncertainty as to the relative difference in strength of the bar sizes.

BCC alloys are known to follow a Hall-Petch relationship whereby the yield strength *σ* is proportional to the reciprocal of the square-root of the grain size (*d*). This is given in Equation (2), where *σ_0_* is the friction stress and *k_y_* a proportionality constant [2,3].

(2) $$\sigma =\sigma_{0}+k_{y}d^{-1/2}$$

The value of *k_y_* is important as it quantifies how the strength increases for a given grain size reduction. Dingley and McLean [4] made a theoretical prediction for the relationship between grain size and strength to give a *k_y_* value of 2190 MPa µm^−1/2^, which has been found to show good agreement with the experimental work of a number of different research on steels [4,14,30,31,32,33]. For maraging steels the choice of what size to use is important. Some researchers [6,25] suggest that the main controlling factor in strengthening is the block size and not the PAG size, and find a value of *k_y_* of ~450 MPa µm^−1/2^ using the block sizes. They argue that the PAG size often shows a Hall-Petch relation due to the scaling of block and PAG sizes. Others still [34] have suggested that the controlling size element are dislocation networks with no or small misorientation across them, such as the lath size. In this case the strength from a lath martensite (*σ_L_* in MPa) increases with the reciprocal of the lath size (*d_L_* in units μm):

(3) $$\sigma_{L}=\frac{115\;MPa\;\mu m}{d_{L}}$$

For the Hall-Petch relationship using the block sizes the calculated strength differences between 19 mm and 50 mm samples are approximately 130 MPa and 28 MPa using values of *k_y_* of 2190 MPa µm^−1/2^ and 450 MPa µm^−1/2^ respectively. The higher value of *k_y_* best describes the difference of tensile strength of HT3 at room-temperature (167 MPa), whereas the lower *k_y_* value better describes the differences for HT5 and HT3 at other temperatures. If Equation (3) is used with the lath sizes a lower strength difference is predicted, 10 MPa, than observed.

It is possible to make an estimation of the absolute contribution of strengthening of the lath microstructure by accounting for strengthening from the different elements. For HT3 the intrinsic strength of Fe is taken as 200 MPa [35], solid solution strengthening is taken as 300 MPa [36], and precipitate strengthening is taken as 1020 MPa (from the differences in hardness before after heat-treatment and assuming hardness is directly proportional to the yield strength [37]). This calculation gives a strength of the lath martensite of 610 MPa for HT3 50 mm; this value is close to the strengthening using Equation (2), the block size and a constant of 2150 MPa µm^−1/2^ of 660 MPa, but significantly different to the calculations using the other constant in Equation (2) (~140 MPa) or using Equation (3) (~40 MPa).

Hence, the use of Equation (2) with a constant of 2150 MPa µm^−1/2^ best explains the microstructural contribution to the tensile strength of HT3 for this alloy. Both the block and lath sizes fall with decreasing PAG size; hence it can be difficult to determine which may be causing the strengthening. It is possible that Equation (3) better describes the strengthening, but with a larger constant of proportionality.

At elevated temperatures the value of *k_y_* has been found to fall: between 20 °C and 350 °C the value was said to fall by up to one fifth [30]. This may explain the fall of over one third between the strength of the two bar sizes of HT3 at 450 °C from 20 °C, but not the values at 200 °C.

#### 3.2.2. Elongation to Failure

In contrast to the tensile results both heat-treatments show a marked increase in ductility for the smaller PAG size across all temperatures. At room temperature this difference is much larger for HT3, the 19 mm bar has a ductility 3.7 times that of the 50 mm bar, than for HT5, where the 19 mm bar has 1.3 times the ductility of the 50 mm bar. With increases in temperature the ductility of all conditions increases. Furthermore, at 450 °C both HT3 and HT5 have the same elongation to failure. Fracture surfaces of the samples after room-temperature and elevated temperature testing are shown in Appendix A.

A feature of maraging steels, and BCC metals in general, is a marked transition from ductile to brittle behaviour with falling temperature [27]. The ductile to brittle transition temperature (DBTT) is due to the high dependence of the stress required to move a dislocation with temperature in BCC metals; if the temperature is low enough this stress can exceed the stress needed to propagate a crack and result in a brittle failure. Although, this would suggest the DBTT to occur at a distinct temperature, in reality it is spread over a range of temperatures. The DBTT can shift to higher temperatures with larger grain sizes or when the movement of dislocations is inhibited, such as in the presence of precipitates [5,38,39]. Both the yield and fracture stresses are expected to increases with the reciprocal of the square-root of the grain size, but the fracture stress is expected to have a greater grain size dependence [2], which is why a smaller grain size results in a lower DBTT even if it increases the yield stress. The elongation values (*ε*) at different temperatures (*T*) are fitted to a sigmoid function to determine the DBTT (*T_DBTT_*):

(4) $$\varepsilon =\varepsilon_{min}+\frac{\varepsilon_{max}}{1+e^{-\beta \left(T-T_{DBTT}\right)}}$$

where, *ε_min_* and *ε_max_* are taken as 1% and 12% to represent the minimum and maximum strains and *β* is a fitting constant used to represent the range of the DBTT region. For the conditions studied the DBTT is spread over several hundred degrees Celsius. From this method, we then get HT3 19 mm = −14 °C, HT3 50 mm = 243 °C, HT5 19 mm = 141 °C and HT5 50 mm = 360 °C. The smaller PAG size reduces the DBTT by approximately 200 °C, with a bigger difference for HT3 than HT5. The difference between the two heat-treatments is smaller with the two 50 mm bars having DBTTs separated by less than 100 °C. A linear dependence on the DBTT (*T_DBTT_*) with the log of the reciprocal of the square-root of the grain size (*d*) has been predicted and found experimentally [5]; as shown in Equation (5), where *C* and *α* are constants. Mild steel *α* has been found to have a value of 110 K μm^−1/2^ [5], 190 K μm^−1/2^ for HSLA steel, and 90 K μm^−1/2^ for a 17CrNiMo6 steel [6].

(5) $$T_{DBTT}=C+\alpha ln\left(d^{-1/2}\right)$$

Using the value of *α* of 190 K μm^−1/2^, we find a difference of DBTT between the 19 mm and 50 mm bar sizes of 32 °C and 94 °C for the PAG and block sizes respectively. These DBTT values are less than half of the changes measured and are significantly closer if the PAG size is used. To obtain the DBTT changes observed the value of *α* would need to be over double this at 400 K μm^−1/2^. The discrepancy could be a feature of this alloy, but could also be due to the use of tensile tests rather than impact toughness tests; since differences in strain rates and stress states can change the DBTT [40].

#### 3.2.3. Creep Testing

**Figure 7:**
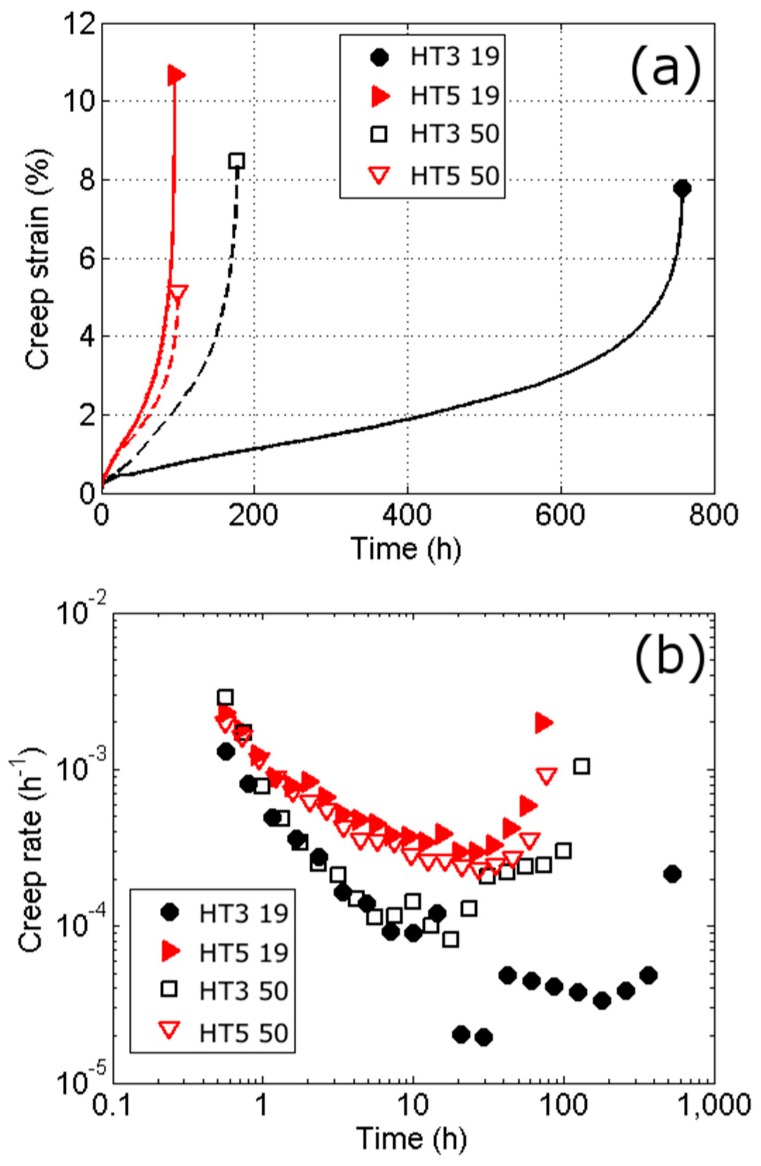
Constant load creep tests at 500 °C at stresses of 0.82 of the yield stress of HT3 at the elevated temperature, and for the different conditions. In (**a**) are the creep curves (plastic strain against time) and in (**b**) the creep rate curves.

In Figure 7 are constant load creep curves and creep rate curves for the different conditions, tested at 500 °C and at a starting stress of 0.82 (the yield stress of HT3 at 500 °C). In a similar manner to the tensile results, the figures show that there is a large difference in properties for the smaller PAG size (19 mm bar) for HT3, but for HT5 the influence of PAG size is much smaller. The two heat-treatments also show the opposite influence of PAG size on the minimum creep rate and final creep strain; for HT3 the smaller PAG size has considerably improved creep properties (four times creep life), whereas for HT5 the larger PAG size has slightly improved properties. For both heat-treatments the difference in creep of the two PAGs occurs at approximately the start of secondary creep; this is shown in Figure 7 where it can be seen that below ~20 h the creep rate appears to be independent of the PAG size.

Although it may be expected that a larger grain size is beneficial for creep, it has often been found that at low to intermediate temperatures, or less than half the melting point of an alloy (*T_m_*) or lower than the lowest temperature for recrystallisation, that a smaller grain size can result in a lower creep rate and a longer time to rupture [7,8,41,42]. In the case of the alloy studied no recrystallisation is expected at 500 °C which is also approximately 0.45 *T_m_*, but it is 0.8 *T_Ae_*_1_ (*T_m_* is the melting point of the alloy and *T_Ae_*_1_ being the equilibrium transformation from martensite to austenite). Although, some differences are expected in the instantaneous strain and primary creep regions [7,8], it is the secondary and tertiary regions where the differences in grain size are expected to be most evident, which is consistent with the findings here. Garofalo et al. [8] derived an expression to describe the change in the steady state creep rate ($\dot{\epsilon_{s}}$) as:

(6) $$\dot{\dot{\epsilon_{s}}=\kappa \left[\frac{2d_{m}^{3}+d^{3}}{d}\right]}$$

where, *d* is the grain diameter, and *κ* a constant. It has been found that at certain temperatures there is an optimum grain size at which the creep rate is minimised, this grain diameter, *d_m_*, falls with increasing temperature. At low enough temperatures *d_m_* can be ignored and the steady state creep rate becomes proportional to the square of the grain size. If it is assumed that the steady state creep dominates, and the strain to failure is independent of time, then from Equation 6 we find that the time to failure is proportional to the reciprocal of the square of the grain size. Both of these relationships have been observed experimentally [7,41,43], which gives the following relations between the steady state creep rate ($\dot{\varepsilon }$) and time to failure (*t*) for two different grain sizes (*d*) 1 and 2 (given by subscripts):

(7) $$\frac{{\dot{\varepsilon }}_{1}}{{\dot{\varepsilon }}_{2}}={\left(\frac{d_{1}}{d_{2}}\right)}^{2}\;\mathrm{and}\;\frac{t_{2}}{t_{1}}={\left(\frac{d_{1}}{d_{2}}\right)}^{2}$$

For each of the equations the term on the right is ~2 when using the block sizes of HT3, or ~4.8 if using the PAG sizes. Whereas, the corresponding terms for the left-hand side of the equations are 2.7 for the strain rate and 4.1 for the time to failure (the drop in strain rate at ~20 h for HT3 19 mm is expected to be due to instrumental effects rather than a material effect and is therefore not taken as the minimum strain rate). The minimum strain rate shows a reasonable correlation with the block size, albeit slightly a higher one than would be predicted from the block sizes. The time to failure values may show more difference due to the additional assumptions on the nature of the creep curve being dominated by steady state creep, which is not exactly the case here. Previous research using this equation has been conducted on FCC alloys with equiaxed grains and the results presented here show there is some justification for the use of Equation (6) for martensitic alloys by using the block size. The results also suggest that at this temperature there is some justification for *d_m_* to be ignored, which would further suggest that additional grain refinement would result in improvements in creep performance.

There is some disagreement in the literature on whether there is a relationship between the grain size and the strain to failure. Wilshire [7] found an increase in the strain to failure with smaller grain sizes. This was attributed to the ability to form a crack of critical length to cause failure becomes more difficult as the grain size falls because the crack has to pass through multiple grains. However, although Shahinian and Lane [41] observed the same relationship at higher temperatures, at lower ones they found a mixed relationship with the total elongation more likely to fall with grain size. With the samples measured here, both behaviours were observed; HT5 showed an increase in elongation for the smaller PAG size and HT3 showed a slight decrease. It is perhaps the case that Wilshire was correct that smaller grains offer a greater resistance to crack propagation and failure but that a longer creep life allows creep damage to accumulate such as to sometimes obscure any relationship between elongation and grain size.

#### 3.2.4. Fatigue Testing

The results of room temperature load control fatigue tests are shown in Table 5. All samples fail by cleavage fracture, with larger cleavage facets for the 50 mm samples reflecting the coarser microstructure (see Appendix A). It is common for the number of cycles to failure of a fatigue test at a particular condition to show a large statistical variation. This can be observed in the data: for HT5 repeats at stress ratios of 0.75 and 0.77 showed significant variations in cycles to failure. This factor along with the limited number of tests performed limits the quantification of the differences between the different conditions. However, even with these considerations there is a clear difference in properties caused by the different PAG sizes. In this case both heat-treatments have better fatigue properties for the smaller PAG size, with both showing improvements in fatigue life of around 80%.

A number of researchers have found that smaller grain sizes are better for fatigue properties [9,10]. The behaviour has been attributed to either a reduction in dislocation pile-ups and internal stresses from the presence of smaller grains, or to an increase in the difficulty of cracks to propagate through the material. There is a similarity in the fracture surfaces of the fatigue and room temperature tensile samples; this and the fact that the elongation and fatigue life are the only two properties which improve for both HTs suggest the mechanisms controlling the failure of both tests are similar.

## 4. Discussion

The martensitic microstructure can have two separate mechanisms by which it influences the mechanical properties: (1) the yield strength and (2) the cleavage strength. The first of these, often called Hall-Petch strengthening, causes an increase in the force needed to move a dislocation because of the ability of a grain boundary to act as a barrier to its motion. It is this mechanism which causes the hardness and strength increase, and creep performance improvements for the smaller PAG sizes. The second mechanism is the stress needed to form a crack large enough to cause failure. And it is this mechanism that is likely to be responsible for the improvements in the elongation to failure, and the lowering of the DBTT, along with the improvements in fatigue properties that are observed for the smaller PAG sized samples. It can be difficult to separate these mechanisms, as in many cases both will change together and some of their original mathematical descriptions were based on the same concept: the stress at the end of a dislocation pile-up [44]. These two mechanisms will also have different contributions depending on the test type. This can be seen by the larger differences in hardness, between the two bar sizes, compared to the tensile strengths.

An interesting feature of the results is that if we perform an extended age, HT5, there is a change in the fracture stress (elongation to failure and fatigue life) with PAG size but not an increase in the yield strength in the creep and room-temperature tensile tests. Whereas, for HT3 both mechanisms contribute to improved properties. There is not an obvious reason to explain these results. To understand the possible causes, we consider the three main differences in HT3 relative to HT5; (1) precipitate population; (2) solute content and (3) martensite microstructure.

During the extended age of HT5 new precipitates form and existing ones grow in size. This causes a considerable increase in the strength of HT5 relative to HT3 of ~300 MPa. The strength of the matrix is increased relative to the grain boundaries which could in turn reduce the build-up of dislocation pile-ups, or of long-range internal stresses, by grain boundaries. Hence, any change in grain boundary strengthening is reduced. Alternatively, since laves precipitates preferentially form on dislocation boundaries then if the laths and blocks are closer the laves precipitates will be too, which will cause a strength increase. After the extended age of HT5 new precipitates are formed which could be situated within the laths reducing the influence of initial precipitates. However, if precipitation effects were the cause of the difference then the lower creep performance of HT5 compared to HT3 is unusual.

One theory on the cause of yield strength changes with grain size is based on the ability of a grain boundary to generate dislocations [8]. If existing dislocations are locked by a cloud of solute atoms, as is thought to often occur in steels, then new dislocations can be created near the boundary at a greater stress than that needed to move free dislocations. This stress is caused by a dislocation pile-up in the neighbouring grain that gives the grain size dependence of the yield strength. There are experimental observations to back this theory as it has been found that when free dislocations are present or are less tightly bound [45,46], or when dislocations are generated at sources other than grain boundaries [47] the grain size yield strength contribution, or *k_y_*, is significantly reduced. HT5 has a reduced content of solute atoms due to the extended age and so it is possible that it is less locked than in HT3 reducing the value of *k_y_*. Alternatively, dislocations could be more easily generated in HT5 than HT3 because of differences in the precipitates. However, this type of behaviour would imply a lower, or more gradual, yield point for HT5 which is not observed.

There are three main equations that can be used to express the strengthening from the martensitic microstructure: (1) The Hall-Petch equation (Equation (2)); (2) Langford and Cohens dislocation cell equation (Equation (3)), and the Taylor equation [48,49]. The first two have been examined earlier in this report and the last relates the dislocation density to the strength. When a martensitic steel is held at elevated temperatures for an extended time the microstructure can change and coarsen as boundaries move and dislocations reorganise. Since HT5 is held for an extended time at elevated temperatures such a change may be expected that would then help explain why the lack of Hall-Petch strengthening in HT5 by mechanisms (1) and (2). But because EBSD measurements show the martensitic microstructure is unchanged between the two heat-treatments this explanation appears to be not valid. Instead there may be changes in the microstructure at scales smaller than measured by EBSD or changes that do not result in orientation differences. Laths may exist with no misorientation across them, and hence not determined by EBSD, that reorganise during the extended age; this could then explain the behaviour using the strengthening mechanism (2). Alternatively, other dislocations that are not part of a boundary (i.e., forest dislocations) may be present in a greater quantity with smaller PAGs but could be reduced by the extended age; this could then explain the behaviour by mechanism (3).

Unlike the strength and creep properties, the fatigue, elongation and DBTT properties are improved for both heat-treatments. PAG and block boundaries are potential weak points as they can allow cracks to propagate relatively easy because of their length and orientation and the presence of brittle laves phase on them (Sun et al., 2016). This weakness is observed in the fracture surfaces of the tensile, fatigue and creep tests that show fracture preferentially along block and PAG boundaries. Hence, the larger the PAG size, and hence the longer the block size, the easier it is for cracks to propagate and for failure to occur. Therefore, because the PAG and block size is the same for both heat-treatments they both show improvements in fatigue and elongation to failure.

## 5. Conclusions

Two different PAG sizes and two heat-treatments were studied for a novel maraging steel. The mechanical properties of tensile strength, elongation to failure, creep rate and life, and fatigue life were measured for the different conditions in order to understand the influence of PAG, block and lath size on mechanical properties. The changes were quantified and it was shown that the alloy behaved in a similar manner to other alloys with an equiaxed grain structure if the block size of the martensite was used instead of the grain size.

All mechanical properties were improved with a smaller PAG size in one heat-treatment: (a) 170 MPa increase in tensile strength by; (b) four times increase in creep life; (c) four times increase in elongation to failure and (d) 80% increase in fatigue life.

However, the tensile strength increase was not observed at elevated temperatures, and for a heat-treatment with an extended age, only the fatigue and elongation to failure improved. This shows the complexity of the influence of the microstructure on the mechanical properties.

The possible causes of the difference between the alloys were discussed and it was suggested that it is due to different strengthening mechanisms being operative for the yield strength and cleavage strength. The cleavage strength is given by the PAG size and block length controls the elongation to failure, ductile to brittle transition and fatigue life. Whereas, the yield strength given by the block size, which determines the creep and tensile properties. It is proposed that some combination of changes, in (a) the precipitate population, (b) the presence of solid solution elements and (c) the recovery of the lath structure, during the extended age acts to reduce the yield strength difference between the different PAG sizes but not the cleavage strength.

## Figures and Tables

**Table 1:** The chemical composition of the maraging steel.

Element	Fe	Ni	Cr	Co	Mo	W	Al
Weight %	68.11	6.99	9.90	8.02	2.75	2.43	1.80

**Table 2:** Linear intercept sizes from EBSD measurements. Sizes from boundaries with misorientations of more than 1°, 5° and 15°. PAG sizes are determined using ARPGE reconstructions. The 15° values are used as block size and the 2° values as lath sizes. All size values are in μm.

Heat-Treatment	Bar Size	1°	Laths: 2°	5°	10°	Blocks: 15°	PAGs
HT3	19	1.15	2.2	4.9	7	7.5	29.1
HT5	19	1.11	2.4	4.7	6.4	6.9	35.2
HT3	50	1.38	2.9	5.7	9.8	10.6	75.8
HT5	50	1.51	2.9	5.4	8.6	9.4	79.6

**Table 3:** The length of boundaries per unit area (μm^−1^ × 10^2^) for the different samples between certain misorientation angles, taken from the data in Figure 4.

Heat-Treatment	Bar Size	5–10°	10–20°	20–50°	50°+
HT3	19	4.3	3.8	6.5	23.2
HT5	19	4.3	3.8	5.9	26.3
HT3	50	4.4	2.3	1.7	17.5
HT5	50	4.2	2.8	1.6	20.9

**Table 4:** The Vickers hardness in HV-20 of the different conditions.

Sample	19 mm	50 mm
HT3	641.3 ± 5.9	480.4 ± 4.5
HT5	705.7 ± 6.9	489.9 ± 13.2

**Table 5:** The results of load control fatigue tests of the different conditions tested at room temperature and different stresses.

Heat Treatment	Bar Size	Ratio of Applied Stress to Yield Stress of HT3 19 mm	Cycles to Failure	Comparison of Bar Size (% Change)
HT3	50 mm	0.77	13,088	
HT3	50 mm	0.75	21,454	
HT3	19 mm	0.77	19,446	49%
HT3	19 mm	0.75	45,097	110%
			Average	80%
HT5	50 mm	0.77	22,241	
HT5	50 mm	0.75	50,312	
HT5	50 mm	0.77	37,287	
HT5	19 mm	0.77	27,659	24%
HT5	19 mm	0.75	74,636	48%
HT5	19 mm	0.77	100,000 ^1^	168%
			Average	80%

^1^ Test was stopped before failure.

**Figure A1:**
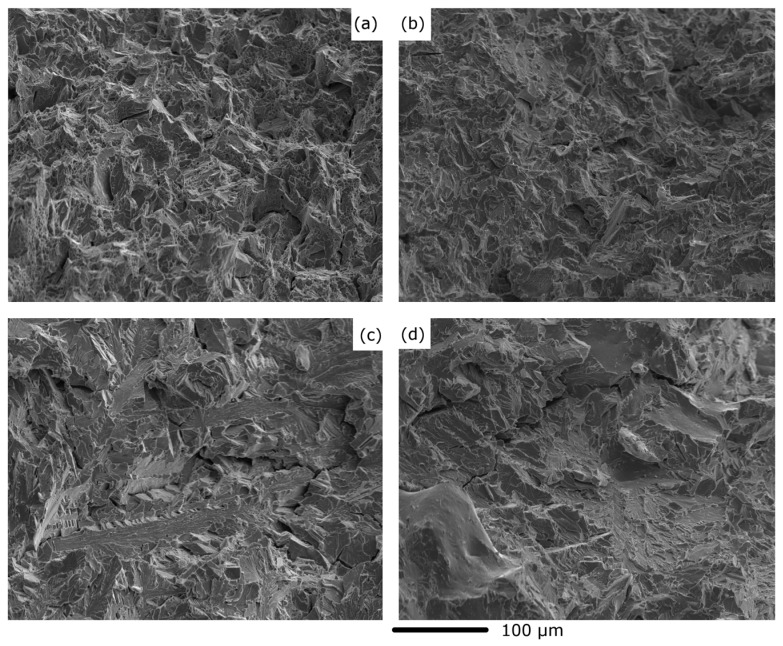
Fracture surfaces of samples after room-temperature tensile tests. (**a**) HT3 19; (**b**) HT5 19; (**c**) HT3 50; (**d**) HT5 50.

**Figure A2:**
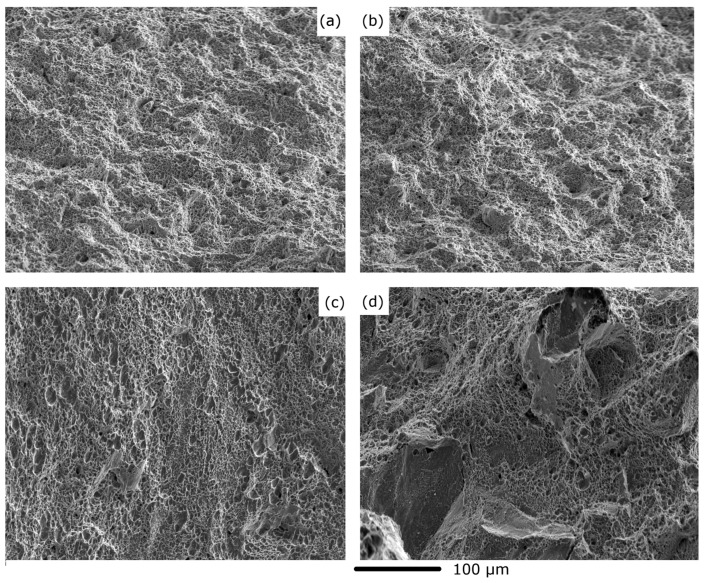
Fracture surfaces of samples after elevated tensile tests at 450 °C. (**a**) HT3 19; (**b**) HT5 19; (**c**) HT3 50; (**d**) HT5 50.

**Figure A3:**
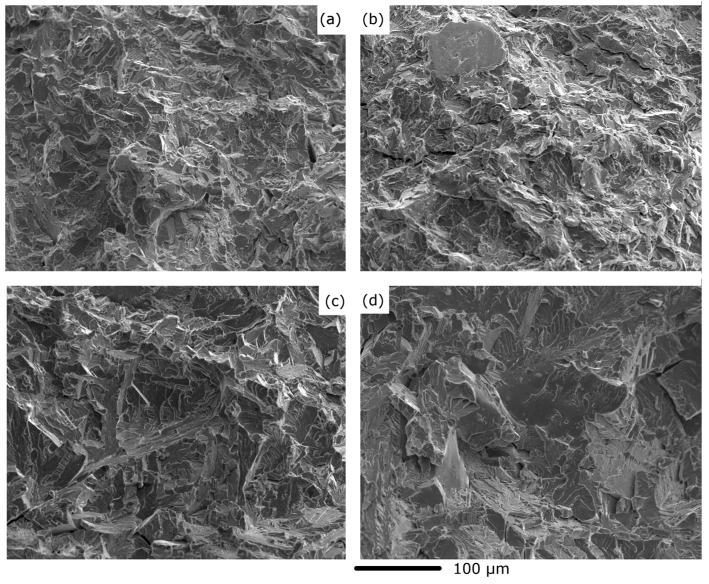
Fracture surfaces of samples after room-temperature fatigue tests, all samples were tested at the same stress. (**a**) HT3 19; (**b**) HT5 19; (**c**) HT3 50; (**d**) HT5 50.

## Appendix A

Selected regions from fractured tensile and fatigue samples are shown in Figure A1, Figure A2 and Figure A3. The fracture surfaces after room-temperature tensile testing (Figure A1) indicate a relatively brittle failure mode, with only a small amount of micro-voids on the 19 mm sample surfaces. The surfaces indicate cleavage fracture, with facets that are larger for the 50 mm samples. After elevated temperature tensile testing (Figure A2), all sample’s fracture surfaces consist of microvoid coalescence and are indicative of a ductile failure. Similar to the room-temperature tests the microvoids are larger for the 50 mm samples. The 50 mm samples also display some quasi-cleavage features. The fatigue fracture surfaces (Figure A3) are like the room-temperature tensile surfaces, and display a cleavage fracture. They also show an increase in facet size with bar sizes.

There are some differences in the fracture surfaces of HT3 and HT5 samples, which are mainly the result of their more brittle failure modes, but the differences are smaller than seen between the different bar sizes.
